# Chemoresistance in Breast Cancer Patients Associated With Changes in P2X7 and A2A Purinergic Receptors in CD8^+^ T Lymphocytes

**DOI:** 10.3389/fphar.2020.576955

**Published:** 2020-11-30

**Authors:** Victor Manuel Ruiz-Rodríguez, Eneida Turiján-Espinoza, Jaime Arturo Guel-Pañola, Mariana Haydee García-Hernández, José de Jesús Zermeño-Nava, Nallely López-López, Sofia Bernal-Silva, Esther Layseca-Espinosa, Ezequiel M. Fuentes-Pananá, Ana María Estrada-Sánchez, Diana Patricia Portales-Pérez

**Affiliations:** ^1^ Translational and Molecular Medicine Laboratory, Research Center for Health Sciences and Biomedicine, Autonomous University of San Luis Potosí, San Luis Potosí, Mexico; ^2^ Central Hospital Dr. Ignacio Morones Prieto, San Luis Potosí, Mexico; ^3^ Unidad de Investigacion Biomédica de Zacatecas, Delegación Zacatecas, Instituto Mexicano del Seguro Social (IMSS), Zacatecas, Mexico; ^4^ Research Unit in Virology and Cancer, Children’s Hospital of Mexico Federico Gómez, Mexico City, Mexico; ^5^ División de Biología Molecular, Instituto Potosino de Investigación Científica y Tecnológica (IPICYT), San Luis Potosí, Mexico

**Keywords:** chemoresistance, P2X7 receptor, A2A receptor, CD8^+^ T cells, obesity, diabetes, BMI

## Abstract

Breast cancer (BRCA) is the most frequent cancer type that afflicts women. Unfortunately, despite all the current therapeutic strategies, many patients develop chemoresistance hampering the efficacy of treatment. Hence, an early indicator of therapy efficacy might aid in the search for better treatment and patient survival. Although emerging evidence indicates a key role of the purinergic receptors P2X7 and A2A in cancer, less is known about their involvement in BRCA chemoresistance. In this sense, as the chemotherapeutic treatment stimulates immune system response, we evaluated the expression and function of P2X7 and A2A receptors in CD8^+^ T cells before and four months after BRCA patients received neoadjuvant chemotherapy. The results showed an increase in the levels of expression of P2X7 and a decrease in the expression of A2A in CD8^+^ T cells in non-chemoresistant (N-CHR) patients, compared to chemoresistant (CHR) patients. Interestingly, in CHR patients, reduced expression of P2X7 occurs along with a decrease in the CD62L shedding and the production of IFN-γ. In the case of the A2A function, the inhibition of IFN-γ production was not observed after chemotherapy in CHR patients. A possible relationship between the modulation of the expression and function of the P2X7 and A2A receptors was found, according to the molecular subtypes, where the patients that were triple-negative and human epidermal growth factor receptor 2 (HER2)-enriched presented more alterations. Comorbidities such as overweight/obesity and type 2 diabetes mellitus (T2DM) participate in the abnormalities detected. Our results demonstrate the importance of purinergic signaling in CD8^+^ T cells during chemoresistance, and it could be considered to implement personalized therapeutic strategies.

## Introduction

Breast cancer (BRCA) is the most common type of cancer in women, both in developed and underdeveloped countries. The World Health Organization (WHO) estimates that 627,000 women died worldwide in 2018 due to this type of cancer. The BRCA incidence rates range from 25.9 cases per 100,000 in South-Central Asia to 94.2 cases per 100,000 in Western Europe (GCO, 2018). In 2015, Mexicos’ BRCA incidence was 27.70 new cases per 100,000 women aged 20 years and older (INEGI, 2016). According to its origin, BRCA can develop in the milk ducts (ductal cancers) or the milk-producing glands (lobular cancers). Biopsy evaluation provides information about the degree of aggressiveness of the tumor and the presence or absence of specific markers that aid in making decisions about the best chemotherapy option. In this sense, patients might fall into one of the four intrinsic molecular groups depending on the presence of estrogen receptor (ER), progesterone (PR) or human epidermal growth factor receptor 2 (HER2): luminal A (ER+, PR+, HER2−), luminal B (ER+, PR+/−, HER2+/−), HER2-enriched (ER−, PR−, HER2+), and triple-negative (ER−, PR−, HER2−) (Breastcancer.org, 2018).

To date, chemotherapy is the most effective treatment for BRCA, which can be adjuvant, applied to patients after mastectomy to avoid a recurrence or neoadjuvant, that targets large tumors to reduce its size before the mastectomy. According to Mexican guidelines, neoadjuvant therapy for BRCA includes fluorouracil, cyclophosphamide, and doxorubicin (Secretaría de Salud, 2017). These drugs favor the death of cancer cells due to their cytotoxic mechanism of action, such as intercalating into DNA, inhibiting pyrimidine synthesis or adding alkyl groups to DNA. In this sense, chemotherapeutic treatment has a stimulating effect on the immune system and its cytotoxic action on the tumor cells (Haynes et al., 2008). One of these effects is observed with doxorubicin or oxaliplatin, where the cancer cells release the alarmin high-mobility group box protein 1 (HMGB1), which acts upon the immune cell patter recognition receptors (PRR). High levels of alarmins are found in patients with BRCA after chemotherapeutic treatment (Apetoh et al., 2007; Stoetzer et al., 2013). On the other hand, doxorubicin induces greater involvement of the CD8^+^ T cells that produce IFN-γ and increases the production of IL-1β and IL-17 in murine models of BRCA. In samples from BRCA patients, higher expression of genes for CD8α, CD8β, and IFN-γ is associated with a better response to treatment with doxorubicin (Mattarollo et al., 2011). However, despite all the available therapeutic strategies, many patients do not adequately respond to chemotherapy and develop chemoresistance (Ji et al., 2019).

ATP also functions as an alarmin and emerging evidence suggests that purinergic signaling pathways play a crucial role in the development of various types of cancer and immune activation (Buxton et al., 2010; Adinolfi et al., 2012; Zhou et al., 2015). In this regard, ATP activation of P2X7 receptors, an ionotropic receptor that belongs to the P2X family, by ATP, promotes the process of metastasis in a BRCA cell line (Xia et al., 2015). In contrast, in glioma patients, higher expression of P2X7 receptors favors radiotherapy's effectiveness by inducing apoptosis (Gehring et al., 2012). The efficiency of anticancer treatment (oxaliplatin, doxorubicin, thapsigargin, staurosporine, or cisplatin) relates to the degree of ATP released by cancerous cells, which promotes the activation of the P2X7 receptor. P2X7 activation contributes to the activation of the NLRP3-inflammasome pathway and the production of IL-1β, favoring antitumor activity, and better treatment efficiency (Ghiringhelli et al., 2009). Therefore, there is conflicting evidence to the prognosis value of P2X7 expression, due to its pro-tumoral or anti-tumoral functions depending its expression in cancer cells or in immune cells.

The A2A and A2B receptors signaling, which belongs to the P1 receptor family, is activated mainly by adenosine, and it also poses immunosuppressive activity. The A2A receptor is the most studied of the P1 receptor family in the regulation of T cell responses. Adenosine is produced from AMP, mainly by CD73, an ecto-5’-nucleotidase. The A2A/A2B receptors antagonists on CD73-positive cancer cells induce a reduction on metastasis while A2A receptor antagonist increases the cytotoxic capacity of the NK cells (Beavis et al., 2013). In triple-negative BRCA (TNBC) that expresses high levels of CD73, conventional chemotherapy is not effective (Spychala et al., 2004; Mittal et al., 2014). In this case, activation of A2B and A2A receptors leads to metastasis and an immune ineffective antitumor activity, which suppresses the efficacy of anthracyclines (doxorubicin), leading to a worse outcome for patients (Loi et al., 2013). These data together suggest that purinergic signaling might influence the efficacy of chemotherapy in BRCA, which might lead to tumor chemioresistance. Because few studies have been conducted with patients, it is crucial to determine the possible involvement of purinergic signaling in chemoresistance mechanisms in BRCA patients and to generate relevant information on the efficacy of first-line drugs to understand better the role of the immune system in the evolution of patients during chemotherapy.

## Material and Methods

### Study Group

This study included 50 newly diagnosed BRCA women that received neoadjuvant chemotherapy with 5-fluorouracil, adriamycin, and cyclophosphamide (FAC) scheme, which is administered for six cycles consisting of one administration every four weeks at doses of 600 mg/m^2^ of five- fluorouracil and cyclophosphamide, and 60 mg/m^2^ of doxorubicin according to the body surface area (BSA) of each patient (Arun et al., 2011). A peripheral venous blood sample was taken before the start of neoadjuvant chemotherapy (C0) and four months later, before administering of the fourth cycle of chemotherapy (C4). The response to chemotherapy was evaluated at the clinic following the Response Evaluation Criteria in Solid Tumors (RECIST), considering the reduction in initial tumor size measured by clinical oncology doctors (Eisenhauer et al., 2009). The patients with a tumor size reduction equal to or less than 30% at C4 were considered chemo-resistant (CHR), including patients who presented tumor enlargement. The molecular identification of each patient’s type of cancer was carried out in the pathology service of the Central Hospital Dr. Ignacio Morones Prieto, San Luis Potosí, using the immunohistochemical or fluorescence *in situ* hybridization (FISH) technique to identify the presence of hormone receptors and HER2; Luminal A (ER+, PR+, HER2−), Luminal B (ER+, PR+/−, HER2+/−), HER2-enriched (ER−, PR−, HER2+), and triple-negative (ER−, PR−, HER2−). All patients signed an informed consent letter before the collection of blood samples. Our study was approved by the Research Committee of the Central Hospital (COFEPRIS 14 CI 24 028 083) and the Committee of Ethics in Research (CONBIOETICA-24-CEI-001-20160427).

### Isolation of Peripheral Blood Mononuclear Cells

The hospital's nursing staff collected the peripheral blood samples during the morning, and patients were no fasting. The blood sample for C0 was collected before the first administration of chemotherapy, and C4 blood was drawn before administering the fourth cycle of chemotherapy. The blood samples were diluted with PBS in a 1:2 ratio and placed in 3 ml of Ficoll per 10 ml of diluted blood. Then, the cells were centrifuged at 2,500 rpm for 20 min, and the fraction of the PBMC was separated and placed in a new tube. Then, a PBS wash was performed at 1,500 rpm for 5 min, and a cell button was obtained and resuspended in RPMI culture medium for the experiments using different stimuli or in PBS, to carry out the analyzes by flow cytometry. Cell viability was determined by trypan blue staining; viable samples were considered when ≥ 95% of living cells were present.

### Flow Cytometry Analysis

The PBMC were placed in a tube (at 2 × 10^5^ cells), washed with PBS, centrifuged at 1,500 rpm for 5 min, and the supernatant was discarded to resuspend the cell button. Surface labeling was performed to determine the expression of P2X7 or A2A receptors on CD8 T cells, incubating the samples with anti-CD8 antibody FITC (BD Bioscience™) or anti-CD8 PE (BD Bioscience™) respectively, for 20 min at 4°C in the dark; the determination of each receptor was performed in separate tubes. After washing with PBS, the cells were fixed with 4% paraformaldehyde (PFA) for 15 min at 4°C in the dark. Subsequently, for P2X7 detection, 0.1% saponin was added for 10 min, then the sample was centrifuged at 1,500 rpm for 5 min, and the supernatant discarded. Rabbit anti-P2X7-human intracellular primary antibody (Sigma-Aldrich) was added and incubated for 30 min at 4°C in the dark. After washing with PBS and discarding the supernatant, the cell button was incubated with the secondary anti-rabbit-PerCP antibody (Santa Cruz Biotechnology) for 30 min at 4°C in the dark. A wash step was then carried out with PBS, and 1% PFA was added and preserved at 4°C. For A2A detection, Triton X-100 was added and incubated for 5 min at 4°C and washed with PBS + 1% albumin at 1,500 rpm for 5 min. The supernatant was discarded, and the cell button was resuspended and incubated with 0.1% saponin for 5 min. Then, the cells were centrifuged at 1,500 rpm for 5 min, and the supernatant discarded. The anti-A2A-PE antibody (Santa Cruz Biotechnology) was added and incubated for 30 min at 4°C in the dark, then 1% PFA was added, and samples were preserved at 4°C. The cells were analyzed in a FACSCanto II flow cytometer (BD Biosciences™) using FlowJo software (Becton, Dickinson and Company). To determine the expression of the P2X7 or A2A receptors in CD8^+^ T lymphocytes by flow cytometry, the following analysis strategy was followed: first, the singlets were selected, and the doublets were discarded with the forward scatter-height (FSC-H) vs. forward scatter-area (FSC-A) analysis. Then, the population of lymphocytes was analyzed based on their size (FSC) and granularity side scatter (SSC), the CD8^+^ T cells were subsequently determined as well as the expression of each receptor.

### Determination of the Shedding of CD62L by Flow Cytometry

The PBMC were incubated in the presence or absence of 100 ng/ml Phorbol 12-myristate-13-acetate (PMA) (as a positive control) or 3 mM ATP at 37°C and 5% CO_2_ for 30 min. The cells were washed with PBS and incubated in the presence of anti-CD8-PE (BD Bioscience™) and anti-CD62L-FITC antibody (BD Bioscience™) for 30 min. After washing with PBS, the cells were analyzed by flow cytometry, and the percentages of CD8^+^ cells that were positive for CD62L were obtained in the presence and absence of ATP. The results are expressed as the percentage of CD62L + cells of CD8^+^ and as the percentage of shedding of CD62L, according to the following formula: percent shedding of CD62L = percentage of CD62L + cells of CD8^+^ without ATP–the percentage of CD62L + cells of CD8^+^ stimulated with ATP.

### Determination of the synthesis of IFN-γ by flow cytometry

The PBMC were incubated in the presence or absence of the CD3/CD28 antibodies (5 µg/ml) for 5 days to favor cell activation at 37°C and 5% CO_2_. Besides, they were incubated separately and simultaneously with the A2A receptor agonist and antagonist, called CGS-21680 (70 µM) and ZM-241385 (10 µM), respectively, these stimuli were used for 1 h before stimulation with the CD3/CD28 antibodies. Cells were washed with PBS and incubated in the presence of an anti-IFN-γ-FITC antibody (BioLegend™) for 30 min. After washing with PBS, the cells were analyzed by flow cytometry.

### Statistical Analysis

The data are shown as the mean ± the standard error of the mean, and the data were analyzed using the GraphPad Prism v.5 program (GraphPad Software Inc., San Diego, CA). The Kolmogorov-Smirnov test determined the distribution of each of the variables. For the analysis of parametric data, a paired Student t-test was performed, and for the case of non-parametric data, a Wilcoxon matched-pairs test was performed. A one-way ANOVA test was performed with a Bonferroni post-hoc test or a Kruskal-Wallis test with Dunn’s post-hoc test to determine the differences between the variables and a Spearman r correlation test to determine the correlation between the variables. A statistically significant difference with a value of P<0.05 was considered.

## Results

### Patient Characteristics

The BRCA study group was 49.9 ± 9.6 years old and presented an average body mass index (BMI) of 30.4 ± 6.7. According to the WHO classification of obesity, 54% of the studied patients presented obesity. Also, 30.9% showed comorbidity with type 2 diabetes mellitus (T2DM), and 31.7% with hypertension. All patients received chemotherapy followed the Mexican guide using FAC therapy (5-fluorouracil, adriamycin, and cyclophosphamide). The patients were classified by the intrinsic molecular type; 36% were luminal A, 24% were luminal B, and 20% of the patients were triple-negative, and HER2 enriched represented a 20% (Table 1).

**TABLE 1:** Anthropometric parameters of the individuals included in this study

Patients	
*n*	50
Age (Years)	49.9 ± 9.6
BMI (Kg/m^2^)	
Before chemotherapy (C0)	30.5 ± 6.6
After chemotherapy (C4)	31 ± 6.6
Normal weight	23.3 ± 1.25 (24%)
Overweight	27.4 ± 1.42 (22%)
Obesity	35.0 ± 5.56 (54%)
T2DM	30.9%
Hypertension	31.7%
Tumor size (cm)	2 × 2–12 × 12
Luminal A (ER+, PR+, HER2−)	36%
Luminal B (ER+, PR+/−; HER2+/−)	24%
HER2-enriched (ER−, PR−, HER2+)	20%
Triple-negative (ER−, PR−, HER2−)	20%
Chemo-resistant	20%

Values are presented as the mean ± standard deviation (SD) or percentage. BMI (body mass index); T2DM (type two diabetes mellitus); estrogen receptor (ER), progesterone receptor (PR) or human epidermal growth factor receptor 2 (HER2).

### Effect of Chemotherapy on P2X7 Expression in CD8^+^ T Cells

**FIGURE 1:**
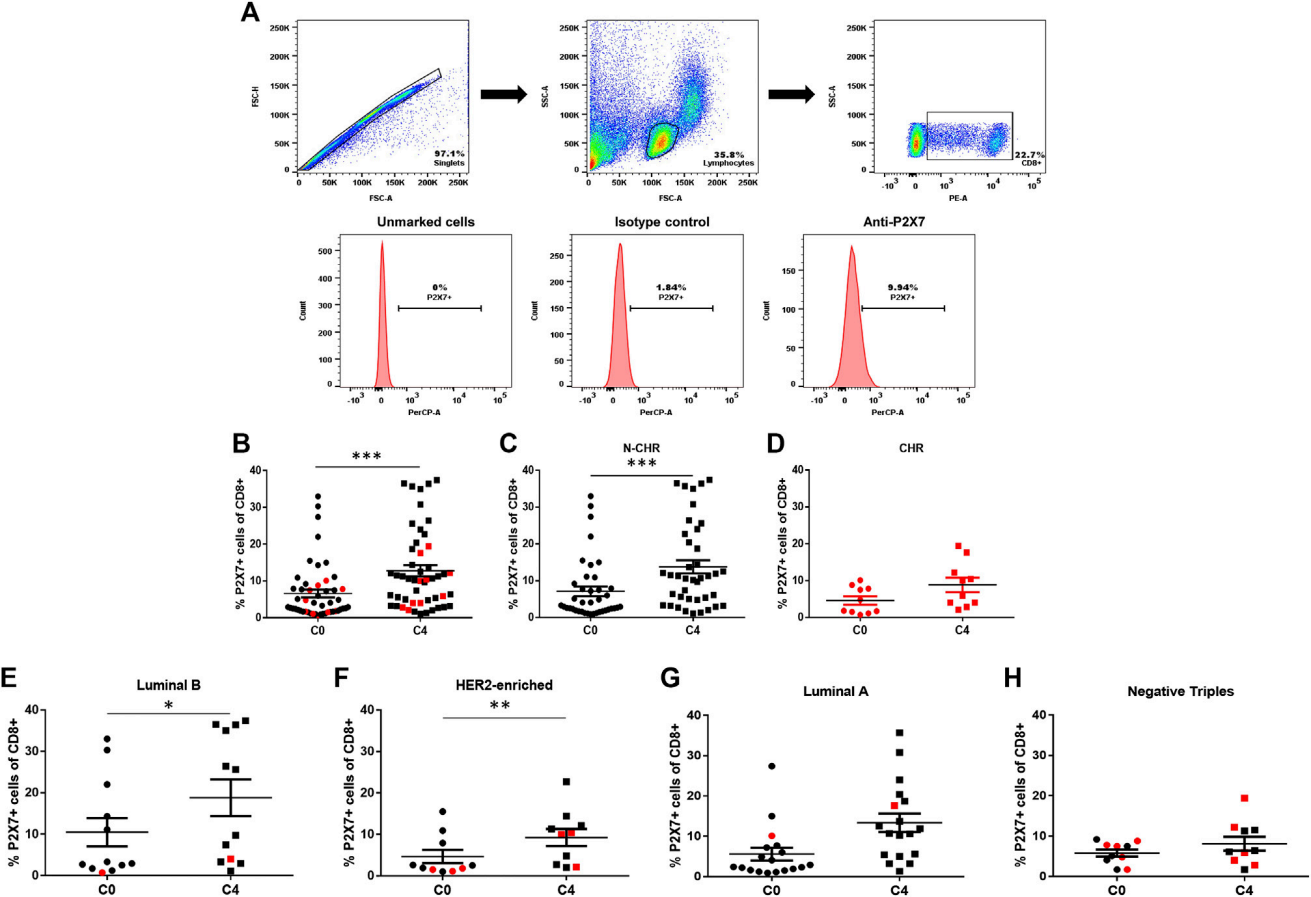
Expression of the P2X7 purinergic receptor in CD8^+^ T cells from Breast cancer (BRCA) patients by flow cytometry. The graphic representations of peripheral blood cells depict the flow cytometry analysis. The singlets were selected, and the lymphocyte population based on cell size and granularity characteristics (FSC and SSC, respectively) was selected. The population of CD8^+^ T cells was determined using specific CD8 staining, and then the expression levels of the P2X7 receptor **(A)** were obtained. Expression levels of P2X7 in CD8^+^ T cells from total samples before the start of neoadjuvant chemotherapy (C0) and four months later, before the administration of the fourth cycle of chemotherapy (C4) **(B)** and non-chemo-resistant (N-CHR) **(C)** or chemo-resistant (CHR) patients **(D)** are shown. P2X7 receptor expression is shown in CD8^+^ T cells in different groups according to their molecular type; luminal A **(E)**, luminal B **(F)**, HER2-enriched **(G)** and triple-negative **(H)** patients. The results correspond to the mean ± SEM. *p<0.05, ***p<0.001. In graphs and onward, CHR is in red while N-CHR is in black symbols.

We assessed how purinergic signaling influences the efficacy of chemotherapy in the BRCA patients, as this pathway has been linked to carcinogenic breast processes. We initially evaluated the expression of P2X7 receptors before and after receiving the FAC chemotherapy (C0 and C4, respectively; see methods). An unmarked control and isotype control were used to finally determine the expression of the P2X7 receptors (Figure 1A). There was a significant increase in P2X7 expression in CD8^+^ T cells at C4 (Figure 1B). However, as 20% of patients developed chemoresistance, we evaluated P2X7 expression within each group. Interestingly, CD8^+^ T cells of non-chemoresistant (N-CHR) patients showed the significant increase in P2X7 positivity (Figure 1C). In contrast, CHR patients showed similar frequencies of P2X7 positive CD8^+^ T cells (Figure 1D). When we evaluated the four intrinsic molecular cancer subtypes, significant increased frequency of P2X7 positivity was observed in luminal B and HER2-enriched BRCA (Figure 1E,F), while no significant changes were detected in luminal A or triple-negative patients (Figure 1G,H).

### Relationship between P2X7 expression, metabolic alterations and tumor size

**FIGURE 2:**
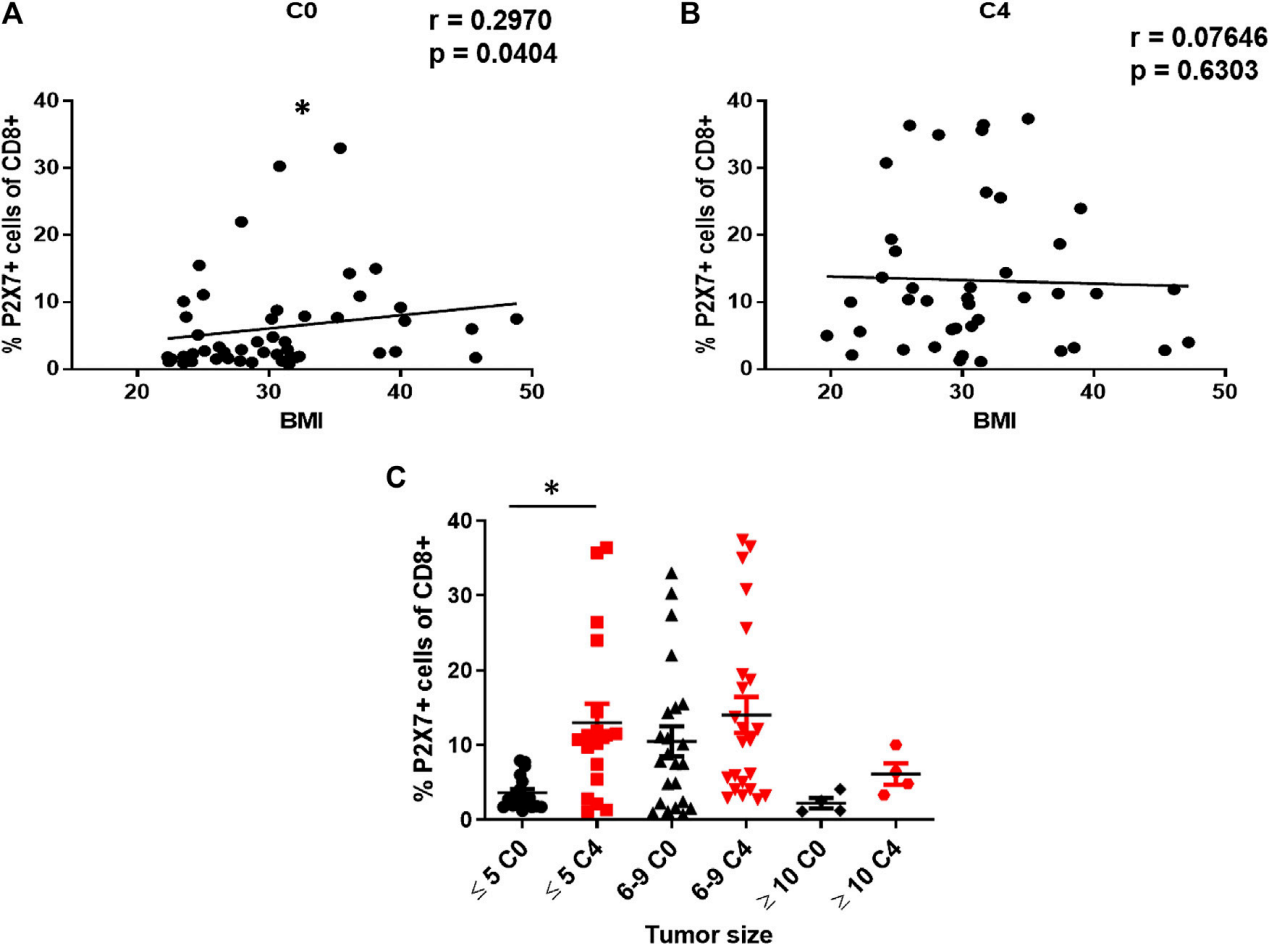
Effect of BMI and tumor size on the expression of P2X7 in CD8^+^ T cells from BRCA patients. Correlation analysis between the expression of the P2X7 receptor and the BMI of patients at C0 **(A)** or C4 **(B)**. Expression of P2X7 in CD8^+^ T cells from patients classified into three groups according to the size of the tumor (≤5 cm, 6–9 cm, ≥10 cm) before starting treatment. Values are shown as the mean ± SEM. *p<0.05.

As 76% of BRCA patients were overweight or obese, we analyzed whether there was a relationship between P2X7 expression in CD8^+^ T lymphocytes and BMI levels. Interestingly, we found a positive correlation before starting chemotherapy (r=0.2970; p=0.04, Figure 2A). However, this correlation was not observed at C4, presumably due to the increase in P2X7 expression in CD8^+^ T cells (r=0.07; p=0.63, Figure 2B); also, there were no changes in the BMI of patients after chemotherapy (see Table 1). Subsequently, when patients were classified depending on their response to chemotherapy, only the N-CHR at C0 showed a correlation between P2X7 expression and BMI (Figure S1B). There was not a correlation between P2X7 and BMI in N-CHR at C4, or CHR at C0 and C4 (Supplementary Figure S1A,B).

As BMI emerged as an influential component of our study, we evaluated P2X7 expression in patients classified accordingly to their BMI (Normal weight ≤ 24.9; Overweight 25-29.9, Obesity ≥ 30), and whether they also presented T2DM. The normal weight and obesity group showed a significant increase in the frequency of P2X7 expression in CD8^+^ T cells in the T2DM group (Supplementary Figure S2A). This difference was maintained only in the group of normal weight when T2DM patients were excluded from the analysis (Supplementary Figure S2B), demonstrating that obesity could play a role in the regulation of P2X7 expression after chemotherapy. On the other hand, when the analysis was performed with BRCA patients who also suffer from T2DM, a significant increase in the expression of P2X7 after chemotherapy in both T2DM and non T2DM (N-T2DM) patients was detected (Supplementary Figure S2C,D), which shows that T2DM has no effect in the regulation of P2X7 expression.

Finally, we explore the change of P2X7 expression in CD8^+^ T cells according to the initial tumors size -the variable that reveals the effectiveness of chemotherapy, as well as disease progression- and a significant increase in P2X7 expression only was observed in patients with initial tumors size ≤ 5 cm (Figure 2C).

### Alterations in the Function of P2X7 in Chemo-Resistant Patients

**FIGURE 3:**
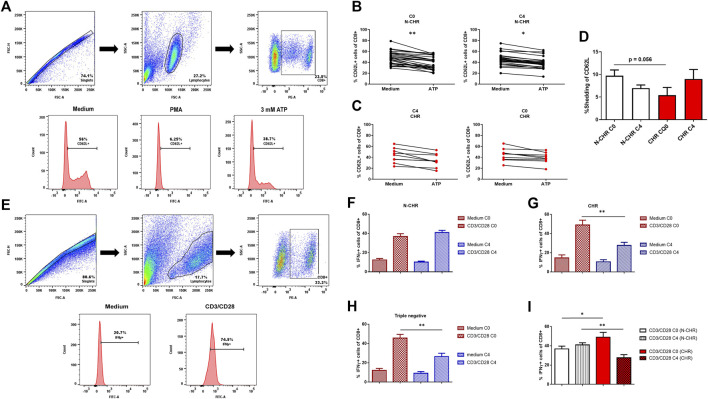
Role of the P2X7 receptor in CD8^+^ T cells from BRCA patients through the shedding of CD62L and the production of IFN-γ. Dot plots representative of the analysis strategy to determine the shedding of CD62L in CD8^+^ T cells are shown. The following different culture conditions were used: RPMI-1640 medium, a positive control, with 100 ng/ml PMA and a condition with 3 mM ATP to stimulate the P2X7 receptor **(A)**. CD62L expression in CD8^+^ T cells at C0 or C4 in N-CHR **(B)** or CHR **(C)** BRCA patients. Comparison analysis of the shedding of CD62L in N-CHR and CHR breast cancer patients at C0 and C4 **(D)**. Dot plots representative of the analysis strategy to determine IFN-γ expression in CD8^+^ T cells under two culture conditions: RPMI-1640 medium and with anti-CD3/CD28 **(E)**. The IFN-γ expression levels in CD8^+^ T cells in the absence or presence of stimulation at C0 and C4 in N-CHR **(F)** or CHR **(G)** patients and triple-negative patients **(H)**. Comparison analysis of IFN-γ expression in CD8^+^ T cells with prior stimulation in N-CHR and CHR patients **(I)**. Values are shown as the mean ± SEM. *p<0.05, **p<0.01.

Next, we determined the function of the P2X7 receptor in CD8^+^ T lymphocytes treated with ATP by flow cytometry measuring two markers of activation, CD62L down-regulation and IFN-γ production. The following analysis strategy was carried out to determine shedding of CD62L. First, the singlets were selected, and the doublets were discarded with the analysis of FSC-H vs. FSC-A. Then, the lymphocyte population was analyzed, considering their size (FSC) and granularity (SSC), in which the population of CD8^+^ T cells and the expression of CD62L was determined (Figure 3A). It is expected that activation of the P2X7 receptor with ATP reduces the expression of CD62L. In N-CHR patients, we found a significant decrease in the frequency of CD62L positive CD8^+^ T cells at C0 and C4 when activating the P2X7 receptor with 3 mM ATP (Figure 3B). In the case of CHR patients, this P2X7 function was lost. No difference in the expression of CD62L was observed in the presence of 3 mM ATP (Figure 3C). The shedding of CD62L calculated at C0 in N-CHR patients was greater than that in CHR patients (Figure 3D); therefore, the activation of P2X7 is diminished in patients with CHR.

The analysis of the P2X7 receptor activation, when divided by the molecular tumor type, showed a significant decrease in the expression of CD62L only in patients classified as luminal A or in ER + patients (Supplementary Figure S3). Regarding the role of P2X7 in patients with metabolic disorders, we only found a significant decrease in the frequency of CD62L positive CD8^+^ T cells in the group of BRCA patients with normal weight or obesity at C0. However, this P2X7 activation was not observed at C4 (Supplementary Figure S4A,B). Additionally, by eliminating the group of patients who also suffer from T2DM to determine the effect of this comorbidity, we were unable to observe any difference (Supplementary Figure S4C,D). In contrast, the analysis of data obtained from T2DM or N-T2DM patients showed a decrease in the levels of expression of CD62L only in the group of N-T2DM patients (Supplementary Figure S4E,F).

The second strategy to study the influence of P2X7 expression in T cell activation was through the production of IFN-γ using the same flow cytometry analysis described above to determine the population of CD8^+^ T cells (Figure 3E). The percentage of IFN-γ producing CD8^+^ T cells was evaluated in a control condition stimulated with anti-CD3/CD28. The N-CHR group presented similar production of IFN-γ between C0 and C4 (Figure 3F). In contrast, the production of IFN-γ was less at C4 in the group of CHR patients (Figure 3G) or triple-negative patients (Figure 3H), compared to C0. Additionally, when comparing the percentage of IFN-γ producing CD8^+^ T cells between N-CHR and CHR patients at C0, we found an increase in the percentage of IFN-γ in CHR vs. N-CHR patients. However, at C4, a significant decrease was observed in the CHR patients compared to N-CHR (Figure 3I).

### Effect of Chemotherapy on A2A Expression in CD8^+^ T Cells

**FIGURE 4:**
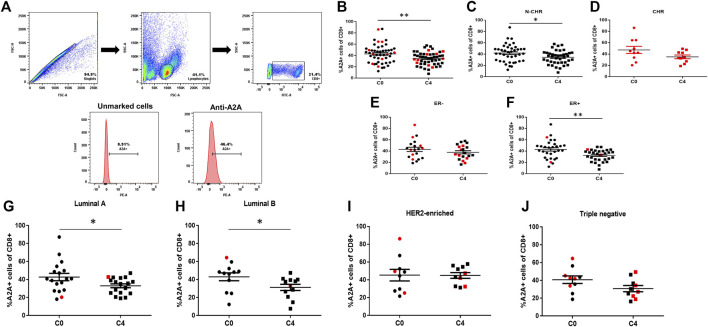
Expression of the A2A purinergic receptor in CD8^+^ T cells from BRCA patients by flow cytometry. Flow cytometric analysis was carried out using the following strategy; the singlets were first selected and then the lymphocyte population, based on cell size and granularity characteristics (FSC and SSC, respectively). The population of CD8^+^ T cells was determined using specific CD8 staining, and the expression levels of the A2A receptor were obtained **(A)**. The A2A expression levels in CD8^+^ T cells of the total samples **(B)**, the group of N-CHR **(C)**, or CHR **(D)** patients. The expression of the A2A receptor in CD8^+^ T cells in different groups according to their molecular type; absence of the estrogen receptor (ER-) **(E)**; presence of estrogen receptor (ER+) **(F)**, luminal A **(G)**, luminal B **(H)**, human epidermal growth factor receptor 2 (HER2)-enriched **(I)**, and triple-negative **(J)**. The results correspond to the mean ± SEM. *p<0.05, **p<0.01.

The expression of the A2A receptor in CD8^+^ T lymphocytes by flow cytometry was determined using an unmarked control as shown in Figure 4A. It has been shown that A2A receptors regulate the antitumor immune response. Contrary to what we observed with the P2X7, we found a significant decrease in the frequency of A2A positive CD8^+^ T cells at C4 compared with the same patient at C0 (Figure 4B). When analyzing the expression of A2A only in N-CHR patients (Figure 4C), a significant decrease was also observed. However, when the CHR patients were analyzed (Figure 4D), no differences were detected. When analyzing A2A expression in the different intrinsic molecular subtypes, we found that A2A positive CD8^+^ T cells decreased significantly at C4 in groups of patients classified as ER+, luminal A, and luminal B patients (Figure 4F–H).

### Relationship Between A2A Receptor in Patients With Metabolic Disorders

When comparing the expression of A2A relative to the BMI, no correlation was found at C0 or C4 (data not shown). However, when A2A expression levels were analyzed in patients with BRCA who also have T2DM, overweight or obesity, we found a significant difference in the expression of A2A between C0 and C4 only in the group of normal-weight patients (Supplementary Figure S5A). Also, by excluding patients who also suffer from T2DM, the significant decrease in A2A expression in patients with normal weight was preserved (Supplementary Figure S5B). On the other hand, if we compared the A2A levels of expression in T2DM (Supplementary Figure S5C) or N-T2DM (Supplementary Figure S5D) patients, we found a significant decrease only in the group of N-T2DM patients.

### Alterations in A2A Function in Chemo-Resistant Patients

**FIGURE 5:**
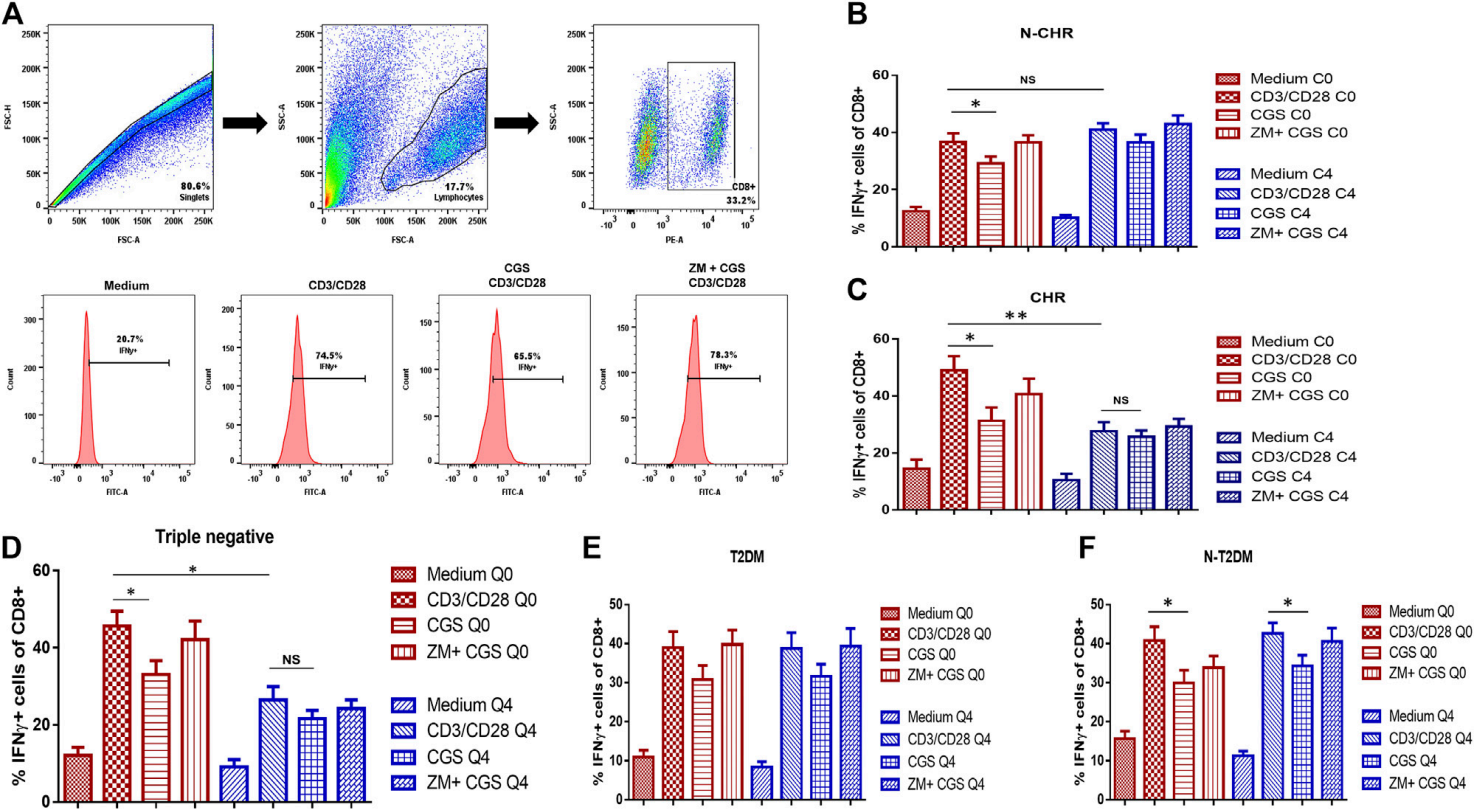
Function of the A2A receptor in CD8^+^ T cells from patients with BRCA by inhibiting IFN-γ production. The analysis strategy to determine IFN-γ expression in CD8^+^ T cells included the following culture conditions: RPMI-1640 medium or pre-stimulation with the CD3/CD28 antibodies. Then, the A2A agonist was added (CGS-21680), or a combination of an agonist and an antagonist (CGS-21680 and ZM-241385) **(A)**. The IFN-γ expression in CD8^+^ T cells at C0 and C4 in N-CHR patients **(B)** or CHR patients **(C)**, triple-negative patients **(D)**, BRCA patients with type 2 diabetes mellitus (T2DM) **(E)**, and N-T2DM patients **(F)**. Values are shown as the mean ± SEM. *p<0.05, **p<0.01.

To determine the function of the A2A receptor, the inhibition in the production of IFN-γ in CD8^+^ T cells was determined by flow cytometry (Figure 5A). The same flow cytometry analysis strategy previously described was followed to determine the percentage of IFN-γ produced by CD8^+^ T cells. It is expected that upon activation of the A2A receptor with CGS-21680, a specific agonist, the percentage of IFN-γ producing by CD8^+^ T cells will decrease and when ZM-241385 -a specific antagonist-is used, the levels of IFN-γ are similar to basal point. A significant decrease in the percentage of IFN-γ producing CD8^+^ T cells at C0 was observed in N-CHR patients when stimulating the A2A receptor. Furthermore, no differences were detected between C0 and C4 when comparing the percentage of IFN-γ producing CD8^+^ T cells (Figure 5B). On the other hand, in the group of CHR patients, a decrease in the percentage of IFN-γ producing CD8^+^ T cells was observed at C0 and C4 when pre-stimulated with CD3/CD28. However, no difference was found in the percentage of cells when stimulating the A2A receptor at C4, which was observed at C0 (Figure 5C). Similar results in CHR patients were observed in the group of patients classified as triple-negative (Figure 5D).

Additionally, we performed an analysis of the A2A receptor function in patients with T2DM. BRCA patients with T2DM did not show a decrease in the percentage of IFN-γ producing CD8^+^ T cells when stimulating the A2A receptor (Figure 5E). The function of A2A was detected only in N-T2DM patients (Figure 5F).

### P2X7+/A2A + Ratio in CD8 T Cells of Breast Cancer Patients

**FIGURE 6:**
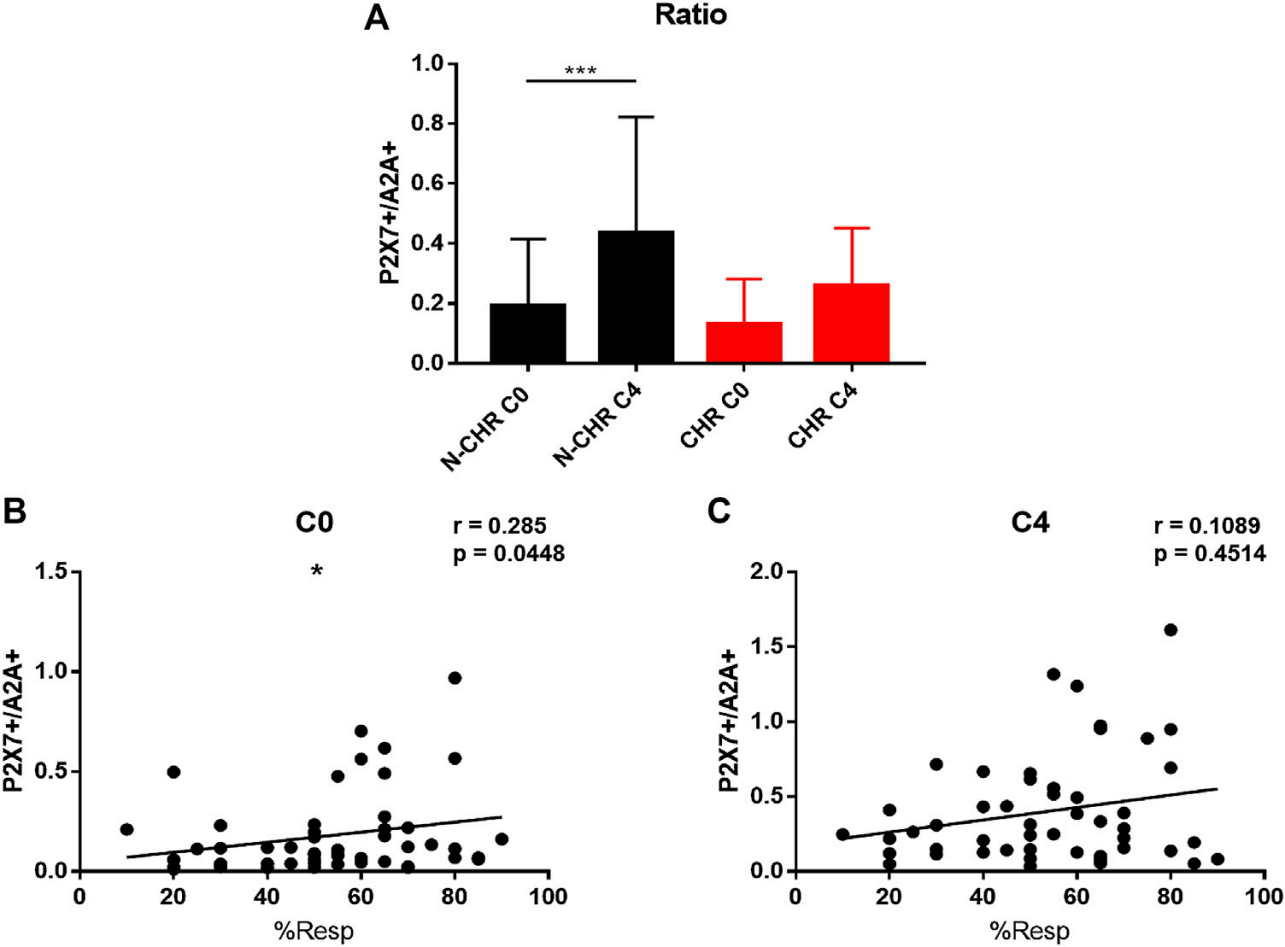
P2X7/A2A ratio in CD8^+^ T cells from patients with BRCA. Analysis of the P2X7/A2A ratio in CD8^+^ T cells from N-CHR and CHR patients at C0 and C4 **(A)**. Correlation analysis between the percentage of response and the P2X7/A2A ratio at C0 **(B)** and C4 **(C)**. Values are shown as the mean ± SEM. *p<0.05, ***p<0.001.

The expression and function results suggest a key role of the P2X7 and A2A receptors in CD8^+^ T cells from patients with BRCA. Then, we analyzed the P2X7/A2A ratio, and a significant increase only in N-CHR patients between C0 and C4 was detected (Figure 6A). Finally, the correlation between the P2X7/A2A ratio and the percentage of response of each patient was determined, where at C0 a positive and significant correlation was obtained (p = 0.0448, R = 0.285, Figure 6B), but at C4 was lost (p = 0.4514, R = 0.1089, Figure 6C).

## Discussion

A growing body of evidence indicates that purinergic signaling plays a key role during tumorigenesis and tumor growth (Di Virgilio and Adinolfi, 2017). Among the purinergic receptors, the P2X7 receptor is one of the most studied as it promotes cancer cell proliferation, angiogenesis, and migration (Di Virgilio and Adinolfi, 2017; Di Virgilio et al., 2018). Besides, the P2X7 receptor participates in immune cells activation response, differentiation, and production of proinflammatory cytokines, and it promotes cell death by forming a non-selective pore, mainly in cells with high expression of the receptor as monocytes/macrophages (Surprenant and North, 2009; Di Virgilio et al., 2017; Adinolfi et al., 2018). Therefore, it is of great interest to determine whether the P2X7 receptor expressed in CD8^+^ T lymphocytes plays a role in the chemoresistance of BRCA patients. Here, we showed that CD8^+^ T cells present an increase in P2X7 receptor expression and a decrease in the A2A content only in patients that positively responded to neoadjuvant chemotherapy. In contrast, patients that developed chemoresistance showed no change in the expression of P2X7 and A2A in CD8^+^ T cells.

Our results showed that the administration of first-line neoadjuvant chemotherapy significantly increased the expression of P2X7 in CD8^+^ T cells. This effect might be caused by the release of molecules such as HMGB1 or ATP from cancer cells treated with drugs used in chemotherapy, which then activate dendritic cells to promote the presentation of an antigen (Aymeric et al., 2010), causing activation of CD8^+^ T cells and elevated the expression of the P2X7 receptor (Borges da Silva et al., 2018). Therefore, this increase in extracellular ATP causes stimulation of the P2X7 receptor in CD8^+^ T lymphocytes, which further favors its activation. On the other hand, in CHR patients, there was no increase in P2X7 receptor expression, which could indicate that the immune response against the tumor triggered after chemotherapy administration is not optimal. In fact, mouse models with deficient P2X7 expression developed accelerated tumorigenesis, lower infiltration of CD8^+^ T cells into the tumor tissue, and reduced chemotherapy efficiency (Ghiringhelli et al., 2009; Adinolfi et al., 2015). Some isoforms of P2X7 have been reported related to cancer (Adinolfi et al., 2010), which could be participating in the mechanisms of chemotherapy efficacy. However, the relationship of each isoform with chemoresistance in BRCA is still unknown.

We also observed altered function of P2X7 receptor in patients who presented chemoresistance, as evidenced by no change in the percentage of CD62L positive cells from CD8^+^ T cells as well as the lower production of IFN-γ in the presence of ATP. These results might be related to the reduced expression of P2X7 receptors in cells from CHR patients. This observation is consistent with published results showing that changes in the expression of P2X7 alter CD8^+^ T cells maturation and its production of IFN-γ that impairs tumor infiltration (Borges da Silva et al., 2018; De Marchi et al., 2019). The shedding of CD62L is dependent on the activity of P2X7 (Mahnke et al., 2017); therefore, low shedding of CD62L reflects diminished function and expression of the P2X7 receptor. In the case of CHR patients, no significant change in the expression of CD62L occurred when stimulated with ATP, as well as low shedding of CD62L at C0. Therefore, these results suggest that an adequate response to chemotherapy largely depends on the level of expression of the P2X7 receptor. However, further assays need to be performed to confirm this hypothesis.

Although it is known that there are differences in the survival of BRCA patients according to the expression of hormonal receptors, such as estrogen, progesterone, or the HER2 receptor (DeSantis et al., 2019), our results showed no changes in the expression of P2X7 and the CD62L marker, as well as low production of IFN-γ in patients classified as triple-negative or HER2-enriched. In the case of luminal B patients, expression levels P2X7 were not modified after chemotherapy, and there were no changes in the CD62L marker. However, the analysis with the data from estrogen receptor-negative (ER−) or positive (ER+) patients showed that only RE–patients presented this alteration in the function of P2X7 (Supplementary Figure S3E,F). Therefore, these results could corroborate previously published information showing a relationship between disease-free survival or overall survival in patients with ER−and triple-negative BRCA (Liu et al., 2012) or HER2+ (Hou et al., 2018) and the presence of CD8^+^ T lymphocytes infiltrated in the tumor, which could indicate that changes in the expression of P2X7 in these BRCA subtypes could be used as a marker of worse prognosis and the survival of these patients.

Metabolic disorders, such as T2DM, overweight obesity, are known as some of the main risk factors that increase the probability of developing cancer (Sun et al., 2017). As our study group was obese or overweight (76%) or had T2DM (30.9%), a significant correlation between P2X7 expression and the BMI of patients before chemotherapy administration was detected, but this correlation was lost during the fourth cycle of chemotherapy. This was possibly due to the side effects of chemotherapy that occasionally produce weight loss. However, when analyzing patients in normal weight, overweight, and obese (excluding patients with T2DM), we observed that in the obese or overweight group, the expression of P2X7 was not modified, and the function was altered. Our results confirm the previously reported relationship between obesity and BRCA, demonstrating the involvement of adipose tissue and the promotion of BRCA metastasis (Sabol et al., 2019). In this sense, we previously reported that purinergic signaling in adipose tissue and peripheral blood participates in generalized low-grade inflammation during overweight or obesity (Ruiz-Rodriguez et al., 2019). Furthermore, BRCA patients with T2DM showed no alteration in the P2X7 expression, which is consistent with previously reported showing that the expression and function of P2X7 on CD8^+^ T cells is not modified in patients with T2DM (Garcia-Hernandez et al., 2011).

On the other hand, one of the variables that are normally considered to determine the progression of cancer, or as an indicator of treatment efficacy, is the tumor size. We found that only patients with a initial tumor size less than or equal to 5 cm presented an increase in the expression of P2X7 in CD8^+^ T cells. These results might indicate a possible relationship between the P2X7 receptor and tumor size, BRCA subtype, and the number of lymphocytes infiltrated in the reported tumor, as was previously reported (Liu et al., 2012; Huang et al., 2015). The possible association between the variations in the percentage of P2X7 in CD8^+^ T cells and chemoresistance, in patients with small initial tumor size (≤ 5 cm), could be a preliminarily indicator of chemoresistance and aid doctors during the decision-making when administering the first-line chemotherapy regimen. In this sense, one question that emerges in the context of our results is how does the expression of the P2X7 and A2A receptors change in the tumor during chemotherapy.

We also evaluated the A2A adenosine receptor, since it participates in the regulation or inhibition of the immune response (Linden and Cekic, 2012; Takenaka et al., 2016). It promotes the development and progression of BRCA tumor cells (Kutryb-Zajac et al., 2018). We found that CD8^+^ T lymphocytes showed a decreased expression of the A2A receptor after the administration of chemotherapy, which may be related to the activation of CD8^+^ T lymphocytes caused by the chemotherapy administration. Dendritic cells play an important role promoting the activation of CD8^+^ T lymphocytes, coupled with an increase in extracellular ATP, possibly causing an increase in the expression of transcription factors such as NF-kB, which has been described as requiring activation in T lymphocytes for adequate control of tumor development (Barnes et al., 2015). Besides, the activation of NF-kB has a negative regulatory role for the A2A receptor (Zhang and Li, 2018; Silva et al., 2019). It has been shown the anergic CD8^+^ T lymphocytes secrete low IFN-γ production impairs NF-kB signaling (Clavijo and Frauwirth, 2012). These results could explain that the decrease in A2A receptor expression in patients after chemotherapy may be evidence for an adequate response to chemotherapy since CHR patients did not show a significant difference in receptor expression after chemotherapy. Also, we observed no changes in IFN-γ production after chemotherapy administration in N-CHR patients. In contrast, in the case of CHR patients, a decrease in IFN-γ production was detected, which supports the idea that no changes in A2A expression can continue to favor an inhibition in IFN-γ production.

Additionally, the expression of A2A did no change in ER−, HER2-enriched or triple-negative patients. A decrease in IFN-γ production after chemotherapy was observed, which might confirm previous findings showing that triple-negative BRCA patients were resistant to chemotherapy with doxorubicin and expressed the ectonucleotidase CD73 (Loi et al., 2013). The CD73 protein favors an increase in extracellular adenosine concentrations, and A2A receptor activation is involved.

BRCA patients with metabolic alterations comorbidity such as for overweight, obesity, or T2DM, significant changes in the modulation of A2A expression, and possibly in its activity might occur. For example, in the case of T2DM, a lack of function in the A2A receptor was found between C0 and C4, which is consistent with previously published results with T2DM patients showing that the A2A receptor has an altered function and a higher receptor expression compared to healthy people (Guzman-Flores et al., 2015). Another comorbidity, such as obesity, hampers the survival of residual tumor cells in a HER2+ BRCA model (Ecker et al., 2019), which may support the idea that obesity has a negative impact on the response to chemotherapy, where the A2A receptor could have a key role. However, a study focused on low-grade inflammation present in adipose tissue from overweight or obese subjects showed that the A2A receptor does not have an important effect on this condition (Cortes-Garcia et al., 2018), which agrees with our results where we did not observe differences in the function of A2A in patients with alterations related to BMI (data not shown). However, obesity could play an important role in the response at the end of chemotherapy. Therefore, more studies are necessary to focus on patients with obesity after chemotherapy has been concluded, and searching for the role of purinergic receptors on residual tumor cells and the possible clinic relapse.

A ratio analysis between the expression or absence of CD16 in monocytes has been previously studied for some types of cancer such as lymphomas to determine possible biomarkers predictive of survival (Zhang et al., 2020). In our study, the P2X7/A2A ratio is proposed as a good prognosis marker related to chemoresistance, which was found only in N-CHR patients and a correlation with the percentage of response before starting chemotherapy. Therefore, it could be useful for decision-making in the use of the neoadjuvant FAC scheme for patients with BRCA, and it is also proposed as a possible marker of chemoresistance in patients with BRCA.

In conclusion, purinergic signaling of P2X7 and A2A receptors in CD8^+^ T correlates with the response of BRCA patients to chemotherapy, and it can be utilized to implement personalized therapeutic strategies. Also, changes in the content of P2X7 and A2A receptors in CD8^+^ T cells could serve as a sign of good prognosis in response to FAC chemotherapy for patients with BRCA, especially in patients with small tumors (≤ 5 cm) and HER2-enriched or triple-negative patients. The metabolic alterations are a risk factor for cancer, but they also have implications for the chemoresistance of patients.

## Data Availability Statement

All datasets generated for this study are included in the article/Supplementary Material.

## Ethics Statement

The studies involving human participants were reviewed and approved by Research Committee of the Central Hospital (COFEPRIS 14 CI 24 028 083) and the Committee of Ethics in Research (CONBIOETICA-24-CEI-001-20160427). The patients/participants provided their written informed consent to participate in this study.

## Author Contributions

All authors contributed to the study conception and design. Material preparation, data collection, and analysis were performed by Victor Manuel Ruiz-Rodríguez and Eneida Turiján-Espinoza. The first draft of the manuscript was written by Victor Manuel Ruiz-Rodríguez, and all authors commented on previous versions of the manuscript. All authors read and approved the final manuscript. Diana Patricia Portales Perez studied the conception and design, contributed to the interpretation of the results, and drafted and critically revised the manuscript.

## Funding

This work was supported by Consejo Nacional de Ciencia y Tecnología (CONACYT) FOSEC SEP-INVESTIGACIÓN BÁSICA A1-S-26479 to AMES, as well as a doctoral fellowship awarded to VMRR (626561) by CONACYT, México.

## Conflict of Interest

The authors declare that the research was conducted in the absence of any commercial or financial relationships that could be construed as a potential conflict of interest.
