# Supplementary material for: Chemoresistance in Breast Cancer Patients Associated With Changes in P2X7 and A2A Purinergic Receptors in CD8+ T Lymphocytes
Source: Front Pharmacol. 2020 Nov 30;11:576955. doi: 10.3389/fphar.2020.576955 (PMC7750810; doi:10.3389/fphar.2020.576955)
Supplement: Supplementary file 1 [file datasheet1.docx]

# Supplementary Figure captions

**Fig. S1. Correlation analysis between the expression of P2X7 in CD8+ T cells and the BMI of chemo-resistant (CHR) or non-chemo-resistant (N-CHR) BRCApatients.** Correlation between the expression of the P2X7 receptor and BMI at C0 and C4 in patients classified as CHR (A) or N-CHR (B). *p<0.05.

**Fig. S2. Effect of metabolic alterations in the expression of the P2X7 receptor in CD8+ T cells in patients with BRCA.** The P2X7 expression levels in patients with breast cancer and T2DM (A) or N-T2DM (B) at C0 and C4, classified into the following three groups according to their BMI: normal weight, overweight, and obese. Expression of P2X7 in breast cancer patients with T2DM (C) or N-T2DM (D). Values are shown as the mean ± SEM. *p<0.05, **p<0.01.

**Fig. S3. Function of the P2X7 receptor in CD8+ T cells by shedding of CD62L in BRCA patients classified by molecular type.** Comparison analysis between CD62L expression in CD8+ T cells at baseline (medium) or with stimulation with 3 mM ATP at C0 or C4 in patients classified as luminal A (A), luminal B (B), HER2-enriched (C), triple-negative (D), ER– (E), or ER+ (F). *p<0.05, **p<0.01.

**Fig. S4. Role of metabolic alterations in the function of the P2X7 receptor in CD8+ T cells through the shedding of CD62L in BRCA patients.** Comparison analysis between CD62L expression in CD8+ T cells at baseline (medium level) and with stimulation with 3 mM ATP in patients with BRCA and T2DM, which were classified into the following three groups according to their BMI: normal weight, overweight, and obese. The results for patients at C0 (A) or C4 (B) are shown. Comparison analysis between the expression of CD62L in CD8+ T cells in patients with BRCA, excluding the group of patients diagnosed with T2DM. The results for patients at C0 (C) or C4 (D) are shown. Comparison analysis between CD62L expression in CD8+ T cells in breast cancer patients at C0 or C4 with T2DM (E) or N-T2DM (F). *p<0.05.

**Fig. S5. Effect of metabolic alterations in A2A receptor expression on CD8+ T cells from patients with BRCA.** The A2A expression levels in patients with BRCA and T2DM at C0 and C4, classified into the following three groups according to their BMI: normal weight, overweight, and obese (A). The A2A expression in patients with BRCA, but without a diagnosis of T2DM, and also classified according to their BMI (B). Expression of A2A in BRCA patients with T2DM (C) or N-T2DM (D). Values are shown as the mean ± SEM. *p<0.05, **p<0.01.
